# lnc-MRGPRF-6:1 Promotes ox-LDL-Induced Macrophage Ferroptosis via Suppressing GPX4

**DOI:** 10.1155/2023/5513245

**Published:** 2023-08-16

**Authors:** Zhihuan You, Xiaotian Ye, Meihua Jiang, Ning Gu, Caihong Liang

**Affiliations:** ^1^Department of Cardiology, The Affiliated Jiangning Hospital of Nanjing Medical University, Nanjing, China; ^2^Department of Geriatrics, The Affiliated Jiangning Hospital of Nanjing Medical University, Nanjing, China; ^3^Department of Cardiology, Nanjing Hospital of Chinese Medicine Affiliated to Nanjing University of Chinese Medicine, Nanjing, China

## Abstract

**Background:**

Ferroptosis, a newly discovered mode of cell death, emerges as a new target for atherosclerosis (AS). Long noncoding RNAs (lncRNAs) are involved in the regulation of ferroptosis. In our previous study, lnc-MRGPRF-6:1 was highly expressed in patients with coronary atherosclerotic disease (CAD) and closely associated with macrophage-mediated inflammation in AS. In the present study, we aim to investigate the role of lnc-MRGPRF-6:1 in oxidized-low-density lipoprotein (ox-LDL)-induced macrophage ferroptosis in AS.

**Methods:**

Firstly, ox-LDL-treated macrophages were used to simulate macrophage injury in AS. Then, ferroptosis-related biomarkers and mitochondrial morphology were detected and observed in ox-LDL-treated macrophages. Subsequently, we constructed lnc-MRGPRF-6:1 knockdown and overexpression of THP-1-derived macrophages and investigated the role of lnc-MRGPRF-6:1 in ox-LDL-induced ferroptosis. Then human monocytes were isolated successfully and were used to explore the role of lnc-MRGPRF-6:1 in macrophage ferroptosis. Likely, we constructed lnc-MRGPRF-6:1 knockdown and overexpression of human monocyte-derived macrophages and detected the expression levels of ferroptosis-related biomarkers. Then, transcriptome sequencing, literature searching, and following quantitative real-time polymerase chain reaction and western blot were implemented to explore specific signaling pathway in the process. It was demonstrated that lnc-MRGPRF-6:1 may regulate ox-LDL-induced macrophage ferroptosis through glutathione peroxidase 4 (GPX4). Eventually, the correlation between lnc-MRGPRF-6:1 and GPX4 was measured in monocyte-derived macrophages of CAD patients and controls.

**Results:**

The ox-LDL-induced injury in macrophages was involved in ferroptosis. The knockdown of lnc-MRGPRF-6:1 could alleviate ox-LDL-induced ferroptosis in macrophages. Meanwhile, the overexpression of lnc-MRGPRF-6:1 could intensify ox-LDL-induced ferroptosis. Furthermore, the knockdown of lnc-MRGPRF-6:1 could alleviate the decrease of GPX4 induced by RAS-selective lethal compounds 3 (RSL-3). These indicated that lnc-MRGPRF-6:1 may suppress GPX4 to induce macrophage ferroptosis. Eventually, lnc-MRGPRF-6:1 was highly expressed in the monocyte-derived macrophages of CAD patients and was negatively correlated with the expression of GPX4.

**Conclusion:**

lnc-MRGPRF-6:1 can promote ox-LDL-induced macrophage ferroptosis through inhibiting GPX4.

## 1. Introduction

As the increasing incidence of coronary atherosclerotic disease (CAD) with high mortality and disability rate, it has attracted great attention. Atherosclerosis (AS), the pathologic basement of CAD, is worth studying in-depth [1–3]. As is known to all, inflammatory progression is essential basis of AS [4]. Macrophage-mediated inflammation plays an important role in the pathophysiology of AS [5]. The progression of inflammation is closely associated with cell death [6].

Ferroptosis is the newly discovered and unconventional form of cell death in 2012, which is different from the previously reported cell death modes [7]. Ferroptosis is defined as iron-dependent lipid peroxidation process, which is closely related to many diseases [8–10]. The morphologic changes of mitochondria including the reduction of mitochondrial cristae and the increase of mitochondrial outer membrane density were characteristic [11]. Increased iron uptake and iron release from intracellular ferritin autophagy will lead to iron accumulation [12]. The excess accumulation of iron results in the abundance of reactive oxygen species (ROS) through Fenton reaction [13]. ROS exceeding the capacity of reductase can directly promote excessive oxidative stress and accumulation of lipid peroxidation including malondialdehyde (MDA) [14, 15]. Oxidation of polyunsaturated fatty acids (PUFAs) including membrane phospholipids (PLs) is identified as a sign of ferroptosis [7]. The accumulation of lactate dehydrogenase (LDH) and the decrease of cell viability were evident in ferroptosis cells [16]. Meanwhile, antioxidants can effectively reduce the sensitivity to ferroptosis. The synthesis of superoxide dismutase (SOD) and glutathione (GSH) can remove ROS to achieve a balanced state and prevent ferroptosis [17]. Inactivation of cellular antioxidant systems can promote ferroptosis. The amino acid antiporter system Xc^−^/glutathione peroxidase 4 (GPX4) axis is considered as the central inhibition pathway in ferroptosis [18–20]. Solute carrier family 7 member 11 (SLC7A11) is a vital component of system Xc^−^, which can promote the resistance of ferroptosis [21, 22].

Studies showed that ferroptosis is closely involved in AS [12, 23]. It was reported that oxidized-low density lipoprotein (ox-LDL)-treated mouse aortic endothelial cells (MAECs) were involved in ferroptosis and the expression of SLC7A11 and GPX4 reduced significantly. The ferroptosis inhibitor Ferrostatin-1 (Fer-1) could protect against the aggravation of endothelial dysfunction in AS [23, 24]. It was demonstrated that hyperuricemia could promote the formation of ox-LDL-induced macrophage-derived foam cells and promote macrophage ferroptosis significantly in AS through down-regulating nuclear respiratory factor 2 (NRF2)/SLC7A11/GPX4 signaling pathway and Fer-1 could reverse these manifestations [25]. These reports indicate that ferroptosis plays vital role in AS. It is very promising to intervene the progression of AS by regulating ferroptosis.

Long noncoding RNAs (lncRNAs) are a class of nucleotide sequences with a length of more than 200 bases and no protein-coding potential, which play an important role in biological processes such as cell metabolism, proliferation, differentiation, and apoptosis [26]. It is reported that lncRNAs participate in the regulation of ferroptosis. LINC00472, a tumor suppressor modulating expression of P53-, was proven to promote ferroptosis through bounding Ras GTPase-activating protein-binding protein 1 (G3BP1) and upregulating P53 [27]. Moreover, the inhibition of lncRNA plasmacytoma variant translocation 1 (lncRNA PVT1) could suppress ferroptosis through regulating the expression of transferrin receptor (TFRC) and P53 which is associated with ischemia/reperfusion (I/R) closely [28].

Lnc-MRGPRF-6:1, the newly discovered lncRNA by our group which is located on the antisense chain of human chromosome 11, hg38 chr11:69303412-69303807. In our previous studies, it was identified that lnc-MRGPRF-6:1 was significantly increased in plasma of CAD patients and was related to macrophage polarization-mediated inflammation in AS closely. However, the ox-LDL-induced macrophage ferroptosis in AS has not been revealed entirely. This study aims to investigate the role of lnc-MRGPRF-6:1 in ox-LDL-induced macrophage ferroptosis.

## 2. Materials and Methods

### 2.1. Cell Culture and Infection of Lentivirus

THP-1 cells were purchased from Shanghai Institute of Cell Research (Shanghai, China) and incubated with RPMI Medium 1640 (Invitrogen, 11875-093) with 10% fetal bovine serum (Invitrogen, 21985). THP-1-derived macrophages were obtained by inoculating logarithmic growth phase THP-1 cells in six-well plate at 5 × 10^5^/well and incubating with 100 ng/mL phorbol 12-myristate 13-acetate (Beyotime, S1819) for 48 hr. Then, groups were as follows, control group, ox-LDL, ox-LDL + Fer-1, and Erastin group. Macrophages without special treatment were used as the control group. In ox-LDL group, macrophages were incubated with 50 mg/L ox-LDL (Invitrogen, 2188176) for 24 hr. In ox-LDL + Fer-1 group, macrophages were incubated with 50 mg/L ox-LDL and 5 *μ*mol/L Fer-1 (MCE, HY-100579) for 24 hr. In Erastin group, macrophages were incubated with 5 *μ*M Erastin (MCE, HY-15763) for 24 hr to induce ferroptosis. The mitochondrial morphology of macrophages was observed by transmission electron microscopy (TEM). lnc-MRGPRF-6:1 knockdown, overexpression, and negative control lentiviral vectors were purchased from GENECHEM (Shanghai, China). THP-1 cells were infected with different lentiviral vectors, respectively, for 16 hr at multiplicity of infection of 30 to construct lnc-MRGPRF-6:1 knockdown, overexpression, and control cells. Then, 2 mg/mL puromycin (Beyotime, ST551) was used to screen out uninfected cells for 72 hr. RAS-selective lethal compound 3 (RSL-3) was reported as a GPX4 inhibitor. Macrophages were incubated with 5 *μ*M RSL-3 (MCE, HY-100218A) for 24 hr to inhibit the expression of GPX4. All cells were cultured in incubator at 37°C and 5% CO_2_.

### 2.2. Study Population and Isolation of Human Monocyte

In our study, 20 CAD patients and 20 controls were recruited. The inclusion criteria of CAD patients were that any major coronary artery (including left main, left gyrus, left anterior descending, and right main) had ≥50% stenosis as shown by coronary angiography. Another 20 patients with stenosis <50% indicated by coronary angiography were selected as control group. Patients with congenital heart disease, cardiomyopathy, hepatic and renal insufficiency, hematologic diseases, malignant tumor, and other concomitant diseases were all excluded. Ficoll (Solarbio, p8900) was used for the isolation of peripheral blood mononuclear cells (PBMCs) from blood. Then, CD14 microbeads (Invitrogen, 11367D) and magnetic beads conjugated with antibodies against human CD14 were used to separate out monocytes. The identification of human monocytes was performed with CD14 (Servicebio, GB11254) immunofluorescent staining. Subsequently, macrophage colony-stimulating factor (M-CSF) (Beyotime, P5313) was used to induce the formation of human monocyte-derived macrophages at the concentration of 50 ng/mL for 7 days.

### 2.3. Immunofluorescent Staining

The expression of CD14 was assessed by immunofluorescence staining. Human monocytes were fixed in 4% paraformaldehyde (Servicebio, G1101) for 15 min and blocked with 3% bovine serum albumin (Servicebio, GC305010) at room temperature for 30 min. Subsequently, the primary antibody CD14 was used to incubate cells at 4°C overnight. After washed three times with PBS, cells were incubated with Goat Anti-Rabbit IgG (Servicebio, GB21303) at room temperature for 50 min. DAPI (Servicebio, G1012) was used for cellular nuclei staining. Then, all cells were observed with fluorescent microscope (Olympus, Japan).

### 2.4. Biological Indicators Testing

RIPA lysis buffer (Beyotime, P0013B) was used to lyse macrophages. Firstly, the protein concentration in cell lysis supernatant was detected by protein assay kit (Beyotime, P0010S). Then, the MDA assay kit (Beyotime, S0131S), ROS assay kit (Beyotime, S0033S), LDH assay kit (Nanjing Jiancheng BioTEC, A020-2), GSH assay kit (Nanjing Jiancheng BioTEC, A006-1-1), SOD assay kit (Beyotime, S0103), and iron assay kit (Leagene, TC1015) were used to detect the relevant expression in macrophages according to product manual.

### 2.5. Oil Red Staining

Macrophages were stained with oil red staining kit (Solarbio, G1262) and then observed and photographed under the microscope (Olympus, Japan).

### 2.6. Neutral Red Uptake Assay

Macrophages were stained with neutral red staining medium (Beyotime, C0013) according to product manual. Then, the optical density (OD) value was detected using a microplate reader at 540 nm.

### 2.7. Quantitative Real-Time Polymerase Chain Reaction

Total RNA from cells was extracted by RNA Isolation Kit (Vazyme, RC112-01). RNA was reverse transcribed to cDNA with HIScript ⅢRT SuperMix (Vazyme, R323-01). RT-qPCR was performed using ChamQ SYBR qPCR Master Mix (Vazyme, Q341) on Step One Plus Real-Time PCR System (ABI, USA). GAPDH was used as internal reference. Equation 2^−*ΔΔ*Ct^ was used to analyze the relative expression. All the primers were synthesized by GenScript (Nanjing, China). Detailed primer sequences are shown in Table 1.

### 2.8. Western Blot

Lysis buffer (Beyotime, P0013B) containing 1% protease inhibitor (Beyotime, ST505) was used to extract protein. The protein assay kit (Beyotime, P0010S) was used to detect protein concentration. The equal amount of protein was separated by 10% SDS-PAGE gel and transferred to polyvinylidene fluoride membranes. The membranes were blocked with blocking reagent (Beyotime, P0252) for 1 hr followed by incubation with primary antibody GPX4 (CST, #52455), ferritin heavy chain (FTH) (CST, #4393S), and Tubulin (Beyotime, AF1216) at 4°C overnight. Then, the membranes were incubated with HRP-labeled goat anti-rabbit IgG secondary antibody (Beyotime, A0208) for 1 hr at room temperature. The membranes were observed and photographed by Bio-Rad chemiluminescence imaging system after enhanced chemiluminescence solution (Beyotime, P0018S) was exposed. The image gray value was analyzed quantitatively by Image J 1.8.

### 2.9. Cell Counting Kit-8 (CCK-8)

The cell supernatant was replaced with culture medium containing 10 *μ*L CCK-8 detection reagent (Dojindo, CK04) and incubated for 3 hr in incubator and then detected optical density (OD) at 450 nm on the microplate reader (TECAN, Switzerland).

### 2.10. Transcriptome Sequencing and Data Analysis

Total RNA was extracted from lnc-MRGPRF-6:1 knockdown macrophages and control, respectively. Transcriptome sequencing was performed by Beijing Genomics Institute (BGI) (Beijing, China). Analysis of sequencing was carried out with BGI Online platform (http://www.bgionline.cn).

### 2.11. Statistical Analysis

All data were analyzed with SPSS 26.0 (IBM Corp, USA) and GraphPad Prism 9.0 (GraphPad Software, USA). Data were reported as mean ± standard deviation. Quantitative data between the two groups were evaluated using Student's *t*-test. Spearman's correlation analysis was used to calculate correlation. Categorical data were represented by case number (%), and *χ*^2^ test or Yates' correction for continuity was applied. *P* < 0.05 was considered statistically significant difference.

## 3. Results

### 3.1. ox-LDL Induces Ferroptosis in Macrophages

**Figure 1:**
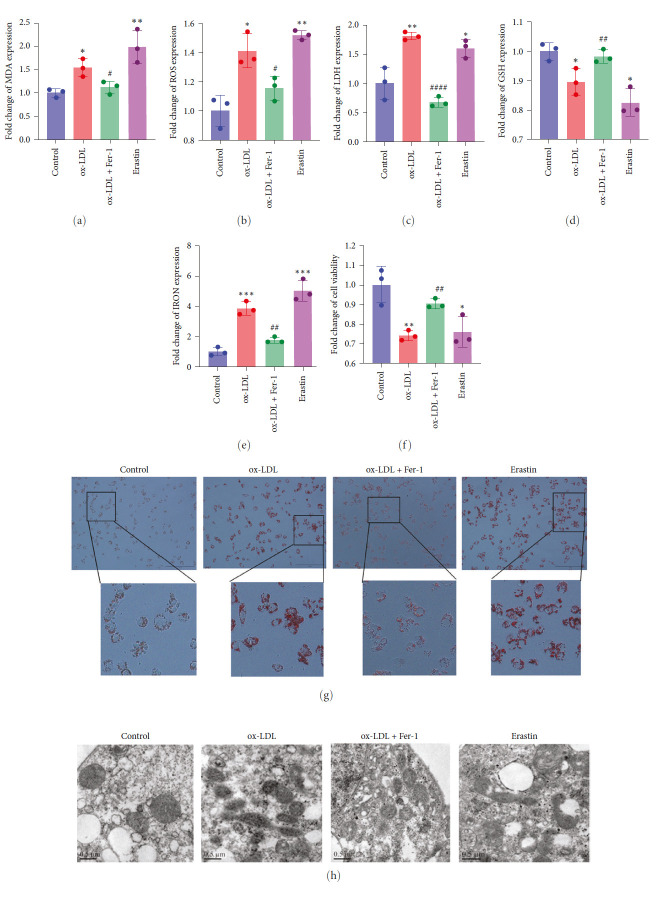
ox-LDL-induced ferroptosis in macrophage. (a–e) The relative expression of MDA (a), ROS (b), LDH (c), GSH (d), IRON (e) in macrophage. (f) Cell viability of macrophage. (g) Images of oil red O staining. (h) Mitochondrial morphology was observed by TEM. Scale bar: 500 nm. The data were expressed as mean with standard deviation (*n* = 3).  ^*∗∗∗∗*^*P* < 0.0001,  ^*∗∗∗*^*P* < 0.001,  ^*∗∗*^*P* < 0.01, and  ^*∗*^*P* < 0.05 vs. control cells. ^####^*P* < 0.0001, ^###^*P* < 0.001, ^##^*P* < 0.01, and ^#^*P* < 0.05 vs. ox-LDL-treated cells. ox-LDL, oxidized-low-density lipoproteins; Fer-1, ferrostatin-1; TEM, transmission electron microscopy. LDH, lactate dehydrogenase; MDA, malondialdehyde; GSH, glutathione; ROS, reactive oxygen species. Control, untreated macrophages; ox-LDL: macrophages treated with 50 mg/L ox-LDL for 24 hr. ox-LDL + Fer-1: macrophages co-treated with 50 mg/L ox-LDL and 5 *μ*mol/L Fer-1 for 24 hr. Erastin: macrophages treated with 5 *μ*M Erastin for 24 hr.

Firstly, we constructed the ox-LDL-treated macrophage model to simulate macrophage injury in AS. Erastin-induced ferroptosis was used as positive control. We measured the expression levels of MDA (Figure 1(a)), ROS (Figure 1(b)), LDH (Figure 1(c)), GSH (Figure 1(d)), IRON (Figure 1(e)), cell viability (Figure 1(f)), and lipid accumulation (Figure 1(g)) in macrophages. Results showed that compared with the control group, the expression levels of MDA, ROS, LDH, iron, and lipid accumulation increased significantly, the expression level of GSH decreased significantly, and cell viability decreased remarkably in the ox-LDL group. Furthermore, the reduction of mitochondrial cristae and the increase of mitochondrial outer membrane density could be observed in macrophage-treated ox-LDL (Figure 1(h)). All the above results were consistent with those of macrophages treated with Erastin. Meanwhile, Fer-1 could partially reverse these results induced by ox-LDL. These indicate that ox-LDL-induced macrophage injury is involved in ferroptosis.

### 3.2. Role of lnc-MRGPRF-6:1 in THP-1-Derived Macrophage Ferroptosis

**Figure 2:**
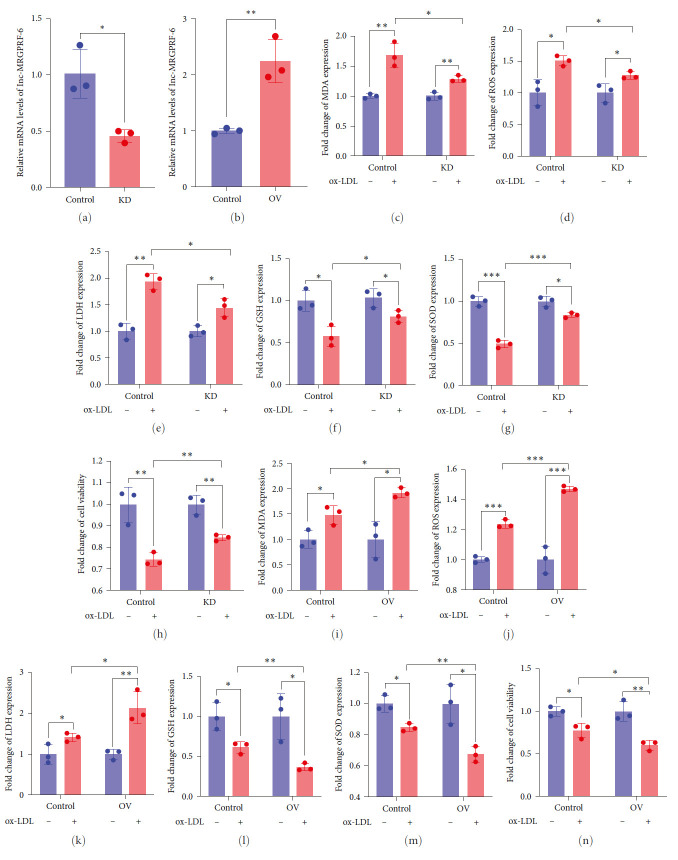
Role of lnc-MRGPRF-6:1 in THP-1-derived macrophage ferroptosis. (a, b) The mRNA level of lnc-MRGPRF-6:1. (c–g, i–m) The relative expression of MDA (c, i), ROS (d, j), LDH (e, k), GSH (f, l), and SOD (g, m). (h, n) Cell viability. The data were expressed as mean with standard deviation (*n* = 3).  ^*∗∗∗∗*^*P* < 0.0001,  ^*∗∗∗*^*P* < 0.001,  ^*∗∗*^*P* < 0.01, and  ^*∗*^*P* < 0.05. 50 mg/L ox-LDL was used to stimulate macrophage for 24 hr. Control, negative control lentivirus-infected macrophages; KD, lnc-MRGPRF-6:1 knockdown macrophages; OV, lnc-MRGPRF-6:1 overexpression of macrophages. MDA, malondialdehyde; ROS, reactive oxygen species; LDH, lactate dehydrogenase; GSH, glutathione; SOD, superoxide dismutase.

It was proven that ox-LDL could induce ferroptosis in macrophage, and the expression levels of MDA, ROS, and LDH were obviously increased. Meanwhile, the expression of GSH and SOD was downregulated. To verify the role of lnc-MRGPRF-6:1 in macrophage ferroptosis, we established the lnc-MRGPRF-6:1 knockdown and overexpression of THP-1-derived macrophage model (Figures 2(a) and 2(b)). We further determined the expression of MDA, ROS, LDH, GSH, and SOD in lnc-MRGPRF-6:1 knockdown and overexpression of THP-1-derived macrophage treated with or without ox-LDL. It was revealed that the knockdown of lnc-MRGPRF-6:1 could reduce the growth of MDA, ROS, and LDH (Figure 2(c)–2(e)) induced by ox-LDL. In addition, the reduction of GSH (Figure 2(f)) and SOD (Figure 2(g)) and decrease of cell viability (Figure 2(h)) induced by ox-LDL was decreased after lnc-MRGPRF-6:1 knockdown. Coincidentally, the overexpression of lnc-MRGPRF-6:1 could promote the ox-LDL-induced increase of MDA, ROS, and LDH (Figure 2(i)–2(k)) and further aggravate the decrease of GSH (Figure 2(l)), SOD (Figure 2(m)), and cell viability (Figure 2(n)). These results suggest that lnc-MRGPRF-6:1 can promote ox-LDL-induced ferroptosis in macrophage. Meanwhile, it was demonstrated that the knockdown of lnc-MRGPRF-6:1 could inhibit macrophage phagocytosis, and the overexpression of lnc-MRGPRF-6:1 could promote macrophage phagocytosis (*Supplementary 1*).

### 3.3. Role of lnc-MRGPRF-6:1 in Human Monocyte-Derived Macrophage Ferroptosis

**Figure 3:**
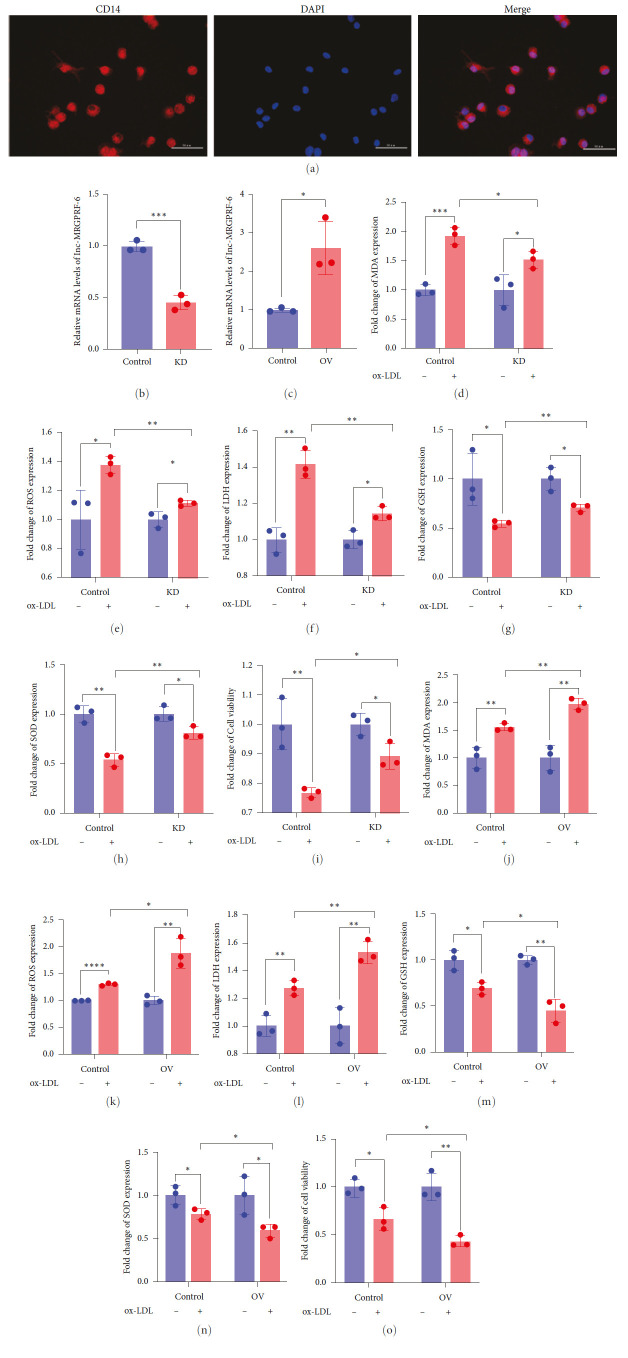
Role of lnc-MRGPRF-6:1 in human monocyte-derived macrophage ferroptosis. (a) CD14 immunofluorescence staining of human monocyte. (b, c) The mRNA level of lnc-MRGPRF-6:1. (d–h, j–n) The relative expression of MDA (d, j), ROS (e, k), LDH (f, l), GSH (g, m), and SOD (h, n). (i, o) Cell viability. The data were expressed as mean with standard deviation (*n* = 3).  ^*∗∗∗∗*^*P* < 0.0001,  ^*∗∗∗*^*P* < 0.001,  ^*∗∗*^*P* < 0.01, and  ^*∗*^*P* < 0.05. 50 mg/L ox-LDL was used to stimulate macrophage for 24 hr. Control, negative control lentivirus-infected macrophages; KD, lnc-MRGPRF-6:1 knockdown macrophage; OV, lnc-MRGPRF-6:1 overexpression of macrophage. MDA, malondialdehyde; ROS, reactive oxygen species; LDH, lactate dehydrogenase; GSH, glutathione; SOD, superoxide dismutase.

Subsequently, we isolated human monocytes and established the human monocyte-derived macrophages. The isolated human monocytes were stained with CD14 (Figure 3(a)). It showed that human monocytes were isolated successfully. Then, we knocked lnc-MRGPRF-6:1 down and overexpressed lnc-MRGPRF-6:1 in human monocyte-derived macrophage (Figures 3(b) and 3(c)). Similarly, the knockdown of lnc-MRGPRF-6:1 could reduce the growth of MDA, ROS, and LDH (Figure 3(d)–3(f)) induced by ox-LDL. The reduction of GSH (Figure 3(g)), SOD (Figure 3(h)), and cell viability (Figure 3(i)) induced by ox-LDL was decreased after lnc-MRGPRF-6:1 knockdown. Coincidentally, the overexpression of lnc-MRGPRF-6:1 could aggravate ox-LDL-induced ferroptosis in human monocyte-derived macrophages (Figure 3(j)–3(o)). These results further suggest that lnc-MRGPRF-6:1 can promote ferroptosis in macrophage. Likewise, it was demonstrated that the knockdown of lnc-MRGPRF-6:1 could inhibit human monocyte-derived macrophage phagocytosis and the overexpression of lnc-MRGPRF-6:1 could promote human monocyte-derived macrophage phagocytosis (*Supplementary 1*).

### 3.4. lnc-MRGPRF-6:1 May Suppress GPX4 to Regulate Macrophage Ferroptosis

**Figure 4:**
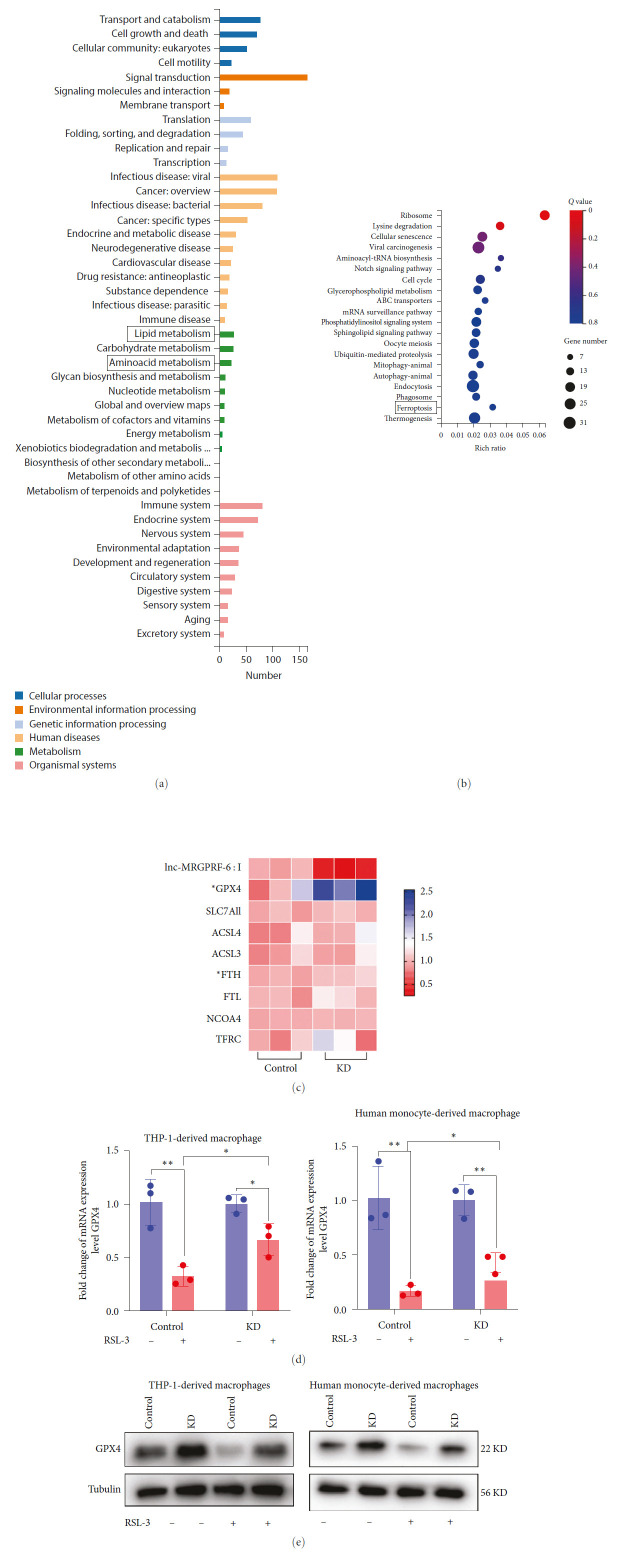
lnc-MRGPRF-6:1 may regulate macrophage ferroptosis via GPX4. (a) KEGG classification analysis between lnc-MRGPRF-6:1 knockdown macrophage and control. (b) KEGG enrichment analysis of between lnc-MRGPRF-6:1 knockdown macrophage and control. (c) The heat map of differential genes involved in ferroptosis between control and KD. (d) The mRNA expression level of GPX4 in macrophage. (e) The protein expression level of GPX4 in macrophage. The data were expressed as mean with standard deviation (*n* = 3).  ^*∗∗∗∗*^*P* < 0.0001,  ^*∗∗∗*^*P* < 0.001,  ^*∗∗*^*P* < 0.01 and  ^*∗*^*P* < 0.05. Macrophages were cultured with 5 *μ*M RSL-3 for 24 hr to inhibit the expression of GPX4. Control, negative control lentivirus-infected macrophage; KD, lnc-MRGPRF-6:1 knockdown macrophage; KEGG: Kyoto Encyclopedia of Genes and Genomes. GPX4, glutathione peroxidase 4; SLC7A11, solute carrier family 7 member 11; ACSL4, Acyl-CoA synthase long-chain 4; ACSL3, Acyl-CoA synthase long-chain 3; FTH, ferritin heavy chain; FTL, ferritin light chain; NCOA4, nuclear receptor coactivator 4; TFRC, transferrin receptor; RSL-3, RAS-selective lethal compounds 3.

To further clarify the signaling pathway, lnc-MRGPRF-6:1 regulates ferroptosis in macrophage. We performed transcriptome sequencing between lnc-MRGPRF-6:1 knockdown macrophages and control. Compared with control, transcriptome sequencing results showed that 668 genes were upregulated and 645 genes were downregulated as the volcano map showed (*Supplementary 2*). Kyoto Encyclopedia of Genes and Genomes (KEGG) pathway classification analysis of differential genes shows that multiple metabolic processes including lipid metabolism and amino acid metabolism which are associated with ferroptosis could be sorted out (Figure 4(a)). Fascinatingly, ferroptosis could be separated out through the KEGG pathway enrichment analysis (Figure 4(b)). NCOA4, TFRC, and ACSL3 were screened out in the transcriptome sequencing results. Lipid metabolism, amino acid metabolism, and iron metabolism are involved in ferroptosis. We supplemented the key genes on the relevant pathways. Combined with literature findings, SLC7A11, GPX4, ACSL3, ACSL4, TFRC, FTH, FTL, NCOA4 were screened out for validation (Figure 4(c)). However, the mRNA expression of SLC7A11, ACSL3, ACSL4, TFRC, FTL, and NCOA4 was not statistically significant between KD and control, the mRNA expression of GPX4 and FTH was statistically significant (Figure 4(c)). Furthermore, the protein expression of GPX4 and FTH was validated. Interestingly, the expression of GPX4 was upregulated accordantly in mRNA and protein level after lnc-MRGPRF-6:1 knockdown. The protein expression of FTH was not statistically significant through western blot validation between KD and control. RSL-3 was used to suppress the expression of GPX4. It was demonstrated that the knockdown of lnc-MRGPRF-6:1 could rescue the reduction of GPX4 induced by RSL-3 (Figures 4(d) and 4(e)). It was indicated that lnc-MRGPRF-6:1 could suppress GPX4 to accelerate ox-LDL-induced macrophage ferroptosis.

### 3.5. lnc-MRGPRF-6:1 Is Highly Expressed in CAD Patients and Is Negatively Correlated with the Expression of GPX4

**Figure 5:**
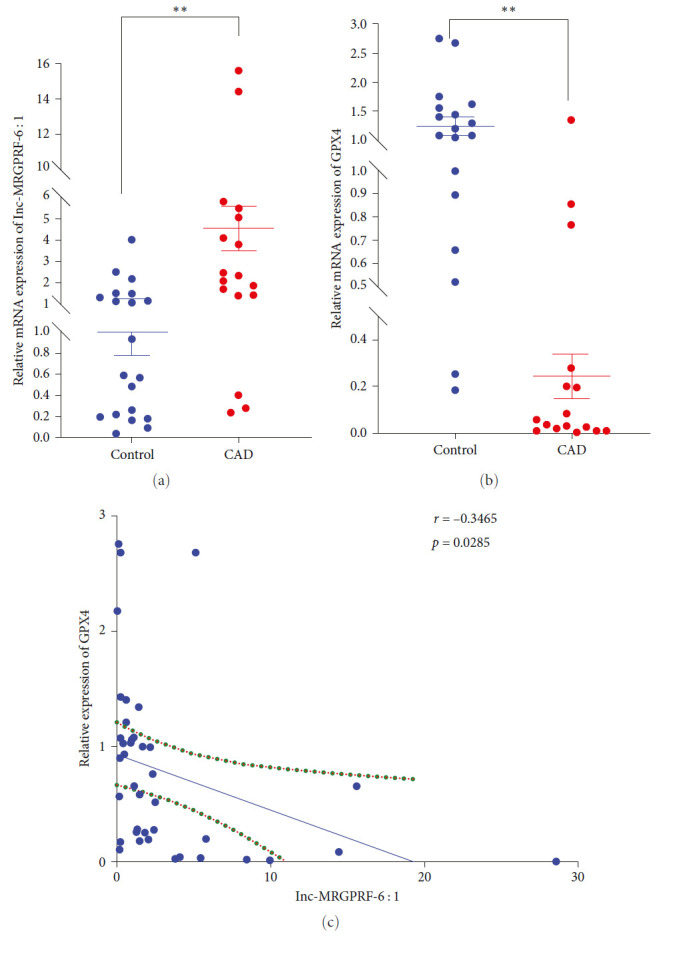
The relative expression and relevance of lnc-MRGPRF-6:1 and GPX4 in the monocyte-derived macrophages of CAD patients and healthy controls. (a) The relative expression of lnc-MRGPRF-6:1 in subjects. (b) The relative expression of GPX4 in subjects. (c) Spearman's correlation analysis of the expression of lnc-MRGPRF-6:1 and GPX4. The data were expressed as mean with standard deviation.  ^*∗∗∗∗*^*P* < 0.0001,  ^*∗∗∗*^*P* < 0.001,  ^*∗∗*^*P* < 0.01, and  ^*∗*^*P* < 0.05 vs. control. CAD (*n* = 20), human monocyte-derived macrophages from patients with coronary artery disease; control (*n* = 20), human monocyte-derived macrophages from controls. GPX4, glutathione peroxidase 4.

Furthermore, 40 subjects were recruited including 20 CAD patients and 20 controls in our study. Clinical and biochemical characteristics of them are shown in Table 2. It was shown that the expression of lnc-MRGPRF-6:1 in CAD patients was significantly higher than those of the control group (Figure 5(a)). In contrast to that, the expression of GPX4 in CAD patients was significantly less than those of the control group (Figure 5(b)). The further Spearman correlation analysis showed that the expression of lnc-MRGPRF-6:1 was negatively correlated with the expression of GPX4 (Figure 5(c)).

## 4. Discussion

The pathophysiological basis of AS is chronic inflammation response involving endothelial cells, macrophages, and smooth muscle cells [29]. Macrophage-mediated inflammation was involved in ferroptosis [30]. The formation of macrophage-derived foam cells participates in the initial stage of AS and plays important role in atherosclerotic plaque formation and plaque rupture [31]. It was reported that the injury of macrophages in AS was associated with ferroptosis [32]. In our previous study, it was demonstrated that lnc-MRGPRF-6:1 was highly expressed in CAD patients and could mediate macrophage polarization to promote inflammation progression in AS [5]. In the present study, it was demonstrated that ox-LDL-induced macrophage ferroptosis and lnc-MRGPRF-6:1 could promote ox-LDL-induced macrophage ferroptosis in AS.

ox-LDL has a great contribution to the formation and rupture of atherosclerotic plaques [33]. Firstly, to verify macrophages treated with ox-LDL were involved in ferroptosis, we established a common macrophage-derived foam cell model with ox-LDL and explored the expression of MDA, ROS, LDH, GSH, and iron and observed the mitochondrial morphology in ox-LDL-treated macrophages. Results indicated that ox-LDL could promote iron accumulation, lipid accumulation, and lipid peroxidation which are consistent with the characteristics of ferroptosis. The most important characteristic of ferroptosis is the variation of mitochondrial morphology. Mitochondrial shrinkage, increased membrane density, reduced mitochondrial cristae and rupture of the outer membrane [7]. Likewise, the mitochondrial cristae reduced and the mitochondrial outer membrane densified in ox-LDL-treated macrophages. Dramatically, all the above induced by ox-LDL could be reversed by Fer-1. These suggested that ferroptosis participates in the formation of ox-LDL-induced macrophage-derived foam cells.

Subsequently, we explored the role of lnc-MRGPRF-6:1 in ox-LDL-induced macrophage ferroptosis. We established the lnc-MRGPRF-6: knockdown and overexpression of macrophage models. Then, the expression of ferroptosis-related biomarkers was assessed. Results show that the knockdown of lnc-MRGPRF-6:1 could reduce ox-LDL-induced lipid accumulation and lipid peroxide. Simultaneously, ox-LDL-induced lipid accumulation and lipid peroxidation were aggravated after lnc-MRGPRF-6:1 overexpression. Similarly, these results could be observed in human monocyte-derived macrophages. It was demonstrated that lnc-MRGPRF-6:1 could promote ox-LDL-induced macrophage ferroptosis.

However, how lnc-MRGPRF-6:1 regulates ox-LDL-induced macrophage ferroptosis remained to be investigated. Transcriptome sequencing was applied to lnc-MRGPRF-6:1 knockdown macrophages and control macrophages. Fascinatingly, lnc-MRGPRF-6:1 was associated with lipid metabolism pathway, amino acid metabolism pathway, and ferroptosis pathway through KEGG pathway classification and enrichment analysis of differential genes. Combined transcriptome sequencing results with literature findings, SLC7A11, GPX4, ACSL3, ACSL4, TFRC, FTH, FTL, NCOA4 were screened out for RT-qPCR validation. SLC7A11 is known as a transmembrane amino acid transporter which can transport extracellular cystine to intracellular and transport intracellular glutamate to extracellular [21, 34, 35]. Intracellular cystine is involved in the synthesis of GSH. GSH cooperated with GPX4 to suppress ferroptosis [36]. GPX4, a unique intracellular antioxidant enzyme, suppresses ferroptosis by neutralizing lipid peroxides and extinguishing lipoxygenase [37, 38]. The inactivation and knockdown of GPX4 generated the accumulation of LPO [39], activated ferroptosis, and further promoted the progression of AS [40, 41]. Likewise, the overexpression of GPX4 could reduce lipid peroxidation and inhibit the progression of AS [42]. Acyl-CoA synthase long-chain family (ACSL) is involved in lipid metabolism pathway. ACSL4 participates in the synthesis of polyunsaturated fatty acids (PUFAs) which can be peroxided easily [43, 44]. It was demonstrated that inactivation of ACSL4 could inhibit ferroptosis obviously. In addition, the overexpression of ACSL4 could promote sensitivity to ferroptosis [45]. However, ACSL3 is involved in the resistance to ferroptosis through increasing the synthesis of monounsaturated fatty acids (MUFAs) which can competitively inhibit the activity of PUFAs [46]. TFRC recognizes the binding of trivalent iron (Fe^3+^) to transferrin to mediate iron transport. TFRC can accelerate iron uptake and ferritin synthesis [47, 48]. As is known to all, ferritin is an important iron storage protein including ferritin heavy chain (FTH) and ferritin light chain (FTL). Increased ferritin synthesis reduces iron accumulation and thus inhibits ferroptosis [49]. Nuclear receptor coactivator 4 (NCOA4) is an important gene that mediates ferritin autophagy. Overexpression of NCOA4 increased iron release through promoting ferritin autophagy. Excess iron accumulation generates ROS in the Fenton reaction and promotes ferroptosis [50]. Fascinatingly, it was demonstrated that the genes with statistic differences were GPX4 and FTH at the mRNA expression level. However, further validation revealed that there was no statistic difference in the protein expression level of FTH, which could be involved in relevant post-transcriptional regulation. Interestingly, western blot validated that GPX4 was still significantly different at the protein level. Furthermore, it was demonstrated that the knockdown of lnc-MRGPRF-6:1 could alleviate RSL-3-induced decrease of GPX4 partly. Accordingly, we speculate that lnc-MRGPRF-6:1 can promote macrophage ferroptosis through inhibiting GPX4.

Eventually, we also investigated the relationship between lnc-MRGPRF-6:1 and GPX4 in subjects. It was demonstrated that lnc-MRGPRF-6:1 was highly expressed in the monocyte-derived macrophages of CAD patients, while the expression of GPX4 was opposite. Furthermore, lnc-MRGPRF-6:1 showed a significant negative correlation with GPX4 through correlation analysis.

In summary, our results clarify that ox-LDL-induced cell damage was involved in ferroptosis. Meanwhile, the ferroptosis inhibitor could alleviate lipid peroxidation and the decrease of cell viability induced by ox-LDL. Furthermore, it is demonstrated that lnc-MRGPRF-6:1 can promote ox-LDL-induced macrophage ferroptosis through inhibiting GPX4. Our findings may contribute to the study of pathophysiology in AS and provide new insights into the treatment of AS.

## Figures and Tables

**Table 1:** Primer sequences in this study.

	Primer sequences
lnc-MRGPRF-6:1 forward	AGGGACAGGAAGATGGTTGGC
lnc-MRGPRF-6:1 reverse	GATGAGCAGAATGGTCGTGAGG
SLC7A11 forward	AACCGAAGGCCAGAGAATCA
SLC7A11reverse	AGGTTCAGGACCTCGAATGG
GPX4 forward	ATACGCTGAGTGTGGTTTGC
GPX4 reverse	CACGCAGATCTTGCTGAACA
ACSL3 forward	AACTGGGATGGCAGAAAGGA
ACSL3 reverse	AGACAGACAAGCTCAGCACT
ACSL4 forward	ATTGGCTACTTGCCTTTGGC
ACSL4 reverse	CAGCCATAAGTGTGGGCTTC
TFRC forward	CATATGTCCCTCGTGAGGCT
TFRC reverse	GCGCTGTCTTTGACCTGAAT
FTH forward	TGAGGAGAGGGAACATGCTG
FTH reverse	TTGTCAGTGGCCAGTTTGTG
FTL forward	CCTGGCCCTAATTTCCTCCA
FTL reverse	AAGCCCTACGGGAAGAGATG
NCOA4 forward	TGGAGCTTGCTATTGGTGGA
NCOA4 reverse	ACATTCCAGGTGACGGCTTA
GAPDH forward	GCGGGGCTCTCCAGAACATC
GAPDH reverse	TCCACCACTGACACGTTGGC

**Table 2:** Clinical and biochemical characteristics of patients with coronary artery disease (CAD) and controls.

	CAD (*n* = 20)	Control (*n* = 20)
Gender (male)	13 (65%)	12 (60%)
Age (years)	64.35 ± 8.16	60.25 ± 7.87
Smoking	13 (65%)	12 (60%)
Drinking	14 (70%)	12 (60%)
BMI (kg/m^2^)	25.64 ± 3.37	25.27 ± 4.03
Hypertension	15 (75%)	13 (65%)
Diabetes	7 (35%)	6 (30%)
WBC (10^9^/L)	6.2 ± 1.72	6.50 ± 1.39
Hemoglobin (g/L)	130.45 ± 10.96	127.7 ± 18.60
Platelet (10^9^/L) ^*∗*^	173.75 ± 47.63	205.65 ± 48.74
ACEI/ARB	6 (30%)	5 (25%)
*β*-Blocker	5 (25%)	3 (15%)
CCB	10 (50%)	12 (60%)
Statin ^*∗∗*^	5 (25%)	4 (20%)
Aspirin	5 (25%)	3 (15%)
Clopidogrel	5 (25%)	1 (5%)
Warfarin	5 (25%)	1 (5%)

*Note*: Categorical data presented as number of total, *n* (%).  ^*∗*^*P* < 0.001,  ^*∗∗*^*P* < 0.05. BMI, body mass index; WBC, white blood cell; ACEI/ARB, angiotensin-converting enzyme inhibitor, angiotensin receptor antagonist; CCB, calcium channel blocker.

## Data Availability

The data used to support the findings of this study are included in the article.

## Supplementary Materials

Supplementary 1 Role of lnc-MRGPRF-6:1 in macrophage phagocytosis.

Supplementary 2 The volcano plot of differential genes from transcriptome sequencing.
