# Supplementary material for: lnc-MRGPRF-6:1 Promotes ox-LDL-Induced Macrophage Ferroptosis via Suppressing GPX4
Source: Mediators Inflamm. 2023 Aug 16;2023:5513245. doi: 10.1155/2023/5513245 (PMC10447047; doi:10.1155/2023/5513245)
Supplement: Supplementary 1 — Role of lnc-MRGPRF-6:1 in macrophage phagocytosis. [file 5513245.f1.pdf]

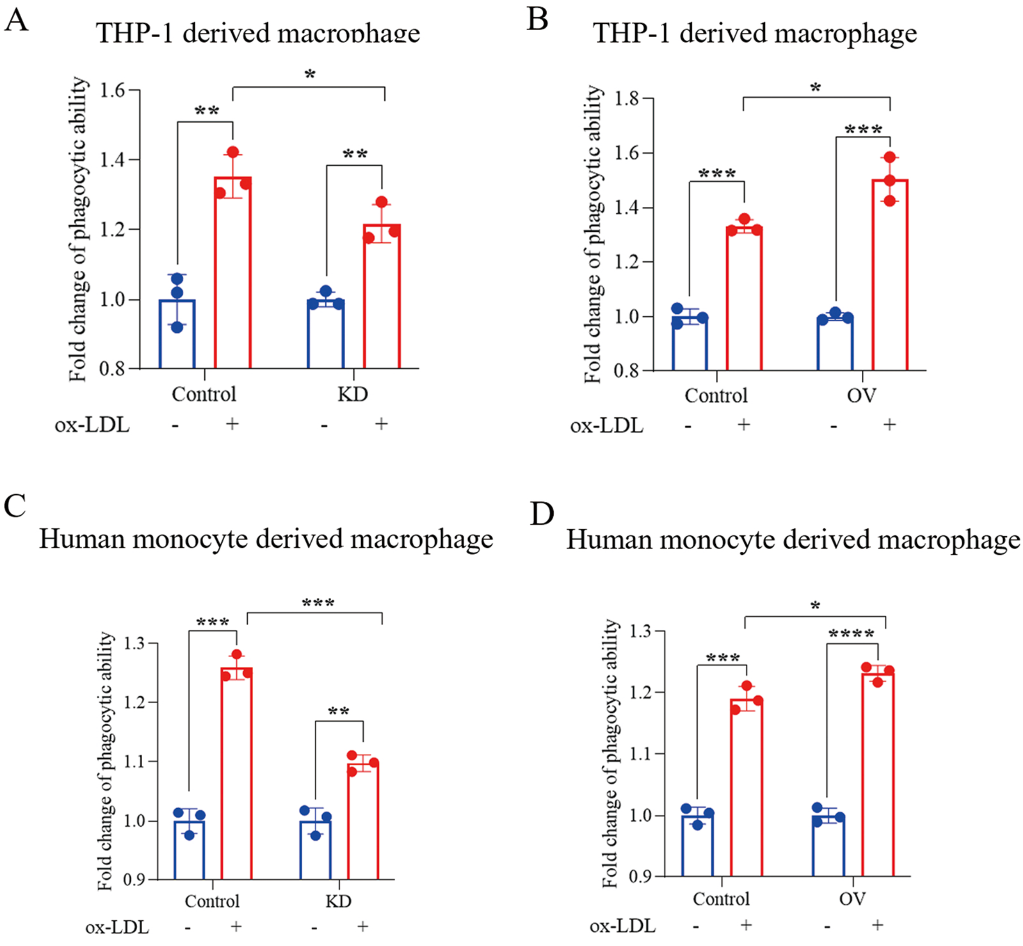
A
THP-1 derived macrophage
Fold change of phagocytic ability
1.6
1.4
1.2
1.0
0.8
*
**
**
Control
KD
ox-LDL
-
+
-
+
B
THP-1 derived macrophage
Fold change of phagocytic ability
1.8
1.6
1.4
1.2
1.0
0.8
*
***
***
Control
OV
ox-LDL
-
+
-
+
C
Human monocyte derived macrophage
Fold change of phagocytic ability
1.3
1.2
1.1
1.0
0.9
***
***
**
Control
KD
ox-LDL
-
+
-
+
D
Human monocyte derived macrophage
Fold change of phagocytic ability
1.3
1.2
1.1
1.0
0.9
*
****
***
Control
OV
ox-LDL
-
+
-
+
