# Supplementary figures and images for: lnc-MRGPRF-6:1 Promotes ox-LDL-Induced Macrophage Ferroptosis via Suppressing GPX4
Source: Mediators Inflamm. 2023 Aug 16;2023:5513245. doi: 10.1155/2023/5513245 (PMC10447047; doi:10.1155/2023/5513245)

Volcano map of DEGs

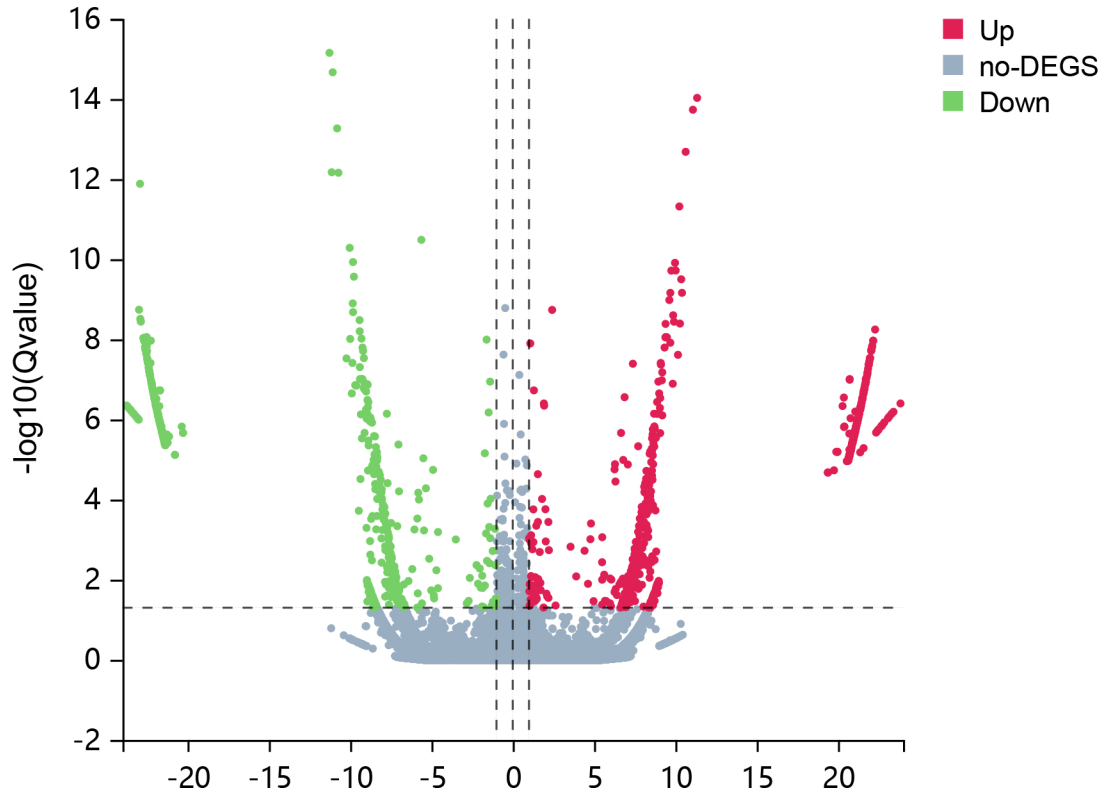

Supplement: Supplementary 2 — The volcano plot of differential genes from transcriptome sequencing. [file 5513245.f2.pdf]
